# Supplementary material for: The Ancient and Evolved Mouse Sperm-Associated Antigen 6 Genes Have Different Biologic Functions In Vivo
Source: Cells. 2022 Jan 20;11(3):336. doi: 10.3390/cells11030336 (PMC8834003; doi:10.3390/cells11030336)
Supplement: Supplementary file 1 [file cells-11-00336-s001.zip › cells-1509682 supplementary.pdf]

```
Spag6 (Chr2)      1     ATGAGCCAGCGGCAGGTGCTGCAAGTGTTCGAGCTGTACCAGAACGCCCGGACCCGGTTT  60
                        |||||||||||||||||||||||||||||||||| ||||||||| |||||||| |  ||
Spag6l (Chr16)    1     ATGAGCCAGCGGCAGGTGCTGCAAGTGTTCGAGCAGTACCAGAAAGCCCGGACGCAATTC  60

Spag6 (Chr2)      61    GTGCAGATGGTGGCGGAGCAGGCGACCAAACCCCAGAACATCGAGACACTGCAGAATGCG  120
                        |||||||||||||| |||| |||||| | |||||| |||||||| || |||||||||||
Spag6l (Chr16)    61    GTGCAGATGGTGGCAGAGCTGGCGACAAGACCCCAAAACATCGAAACGCTGCAGAATGCA  120

Spag6 (Chr2)      121   GGTATAATGTCTTTGCTGAGGCCTCTTCTTCTGGACGTGGTCCCAACCATTCAGCAGACT  180
                        ||| ||||||||||||| ||||| ||||||||||| ||||||||||| ||||| ||||||
Spag6l (Chr16)    121   GGTGTAATGTCTTTGCTAAGGCCGCTTCTTCTGGATGTGGTCCCAACAATTCAACAGACT  180

Spag6 (Chr2)      181   GCTGCCTTGGCTCTGGGGAGGCTGGCCAACTACAACGATGACTTAGCAGAAGCAGTCGTG  240
                        || || |||||||||||||| |||||||| ||||| ||||||||||||||||||||||||
Spag6l (Chr16)    181   GCAGCTTTGGCTCTGGGGAGACTGGCCAATTACAATGATGACTTAGCAGAAGCAGTCGTG  240

Spag6 (Chr2)      241   AAGGGTGACATTCTTCCACAGCTTGTGTATTCATTGGCAGAGCAGAATTGTGTCTACAAG  300
                        || ||||||||||||||||||||||| |||||||||||||| || ||| || | ||||||
Spag6l (Chr16)    241   AAAGGTGACATTCTTCCACAGCTTGTTTATTCATTGGCAGAACAAAATCGTTTTTACAAG  300

Spag6 (Chr2)      301   AAGGCAGCTGCGTTTGTGCTGCGAGCGGTTGGTAAACACTCTCCTCAGCTGGCGCAGGCC  360
                        ||||||||||| |||||| || |||| ||||| |||||||||||||||||||| ||||||
Spag6l (Chr16)    301   AAGGCAGCTGCCTTTGTGTTGAGAGCAGTTGGGAAACACTCTCCTCAGCTGGCCCAGGCC  360

Spag6 (Chr2)      361   ACAGTTGACTGTGGTGCCCTGGACTCACTGGTGATCTGCTTGGAAGATTTTGACCCTGGA  420
                        | ||||||||| || || |||||  ||||||||||||||||||| ||||||||||||||
Spag6l (Chr16)    361   ATAGTTGACTGCGGAGCTCTGGATACACTGGTGATCTGCTTGGAGGATTTTGACCCTGGG  420

Spag6 (Chr2)      421   GTCAAGGAGGCTGCAGCCTGGGCACTTGCATATATTGCAAGGCATAATGCAGAACTGTCG  480
                        |||||||||||||||||||||||||| | ||| |||||||| || ||  ||||| | ||
Spag6l (Chr16)    421   GTCAAGGAGGCTGCAGCCTGGGCACTCGGATACATTGCAAGACACAACACAGAATTATCT  480

Spag6 (Chr2)      481   CAAGCTGTGGTGGATGCGGGAGCTGTTCCTCTTTTAGTCCTCTGTATCCAGGAGCCCGAG  540
                        ||||| ||||||||||| |||||  |||||||||||||||| |||||||||||||| ||
Spag6l (Chr16)    481   CAAGCCGTGGTGGATGCAGGAGCAATTCCTCTTTTAGTCCTTTGTATCCAGGAGCCAGAA  540

Spag6 (Chr2)      541   ACTGCTTTGAAAAGGATCGCTGCCTCGGCCCTCAGTGATATTTCAAAGCACTCCCCAGAG  600
                        | ||||||||||||||||||||||||||||||||||||||| ||||||||||||||||||
Spag6l (Chr16)    541   ATTGCTTTGAAAAGGATCGCTGCCTCGGCCCTCAGTGATATCTCAAAGCACTCCCCAGAG  600

Spag6 (Chr2)      601   TTAGCACAAACGGTGGTGGATGTAGGAGCTATTGCTCACTTAGCCCAGATGATCCTCAAC  660
                        |||||||| ||||||||||| | |||||||||||||||||||||||||||||||||||||
Spag6l (Chr16)    601   TTAGCACAGACGGTGGTGGACGCAGGAGCTATTGCTCACTTAGCCCAGATGATCCTCAAC  660

Spag6 (Chr2)      661   CCCGATGAGAAACTGAAGCAACAGGTCCTCTCAGCTCTGAGTCACATCGCGAAGCACTCG  720
                        || ||||  ||||||||||  ||||||||||||||||| ||||| || || ||||| ||
Spag6l (Chr16)    661   CCTGATGCAAAACTGAAGCGCCAGGTCCTCTCAGCTCTCAGTCAGATTGCAAAGCATTCT  720

Spag6 (Chr2)      721   GTGGACCTGGCAGAGATGGTTGTGGAGGCAGAGATTTTCCCAGTTGTTCTCACCTGTCTG  780
                        |||||| |||||||||||||||| || |||||||||||||| ||||| || ||||| |||
Spag6l (Chr16)    721   GTGGACTTGGCAGAGATGGTTGTTGAAGCAGAGATTTTCCCTGTTGTACTAACCTGCCTG  780

Spag6 (Chr2)      781   AAGGACAAAGATGACTTCGTGAAGAAGAATGCTTGTACTCTGATTAGAGAGATCGCAAAA  840
                        |||||||||||||| |  |||||||| |||||||| ||  | || |||||||| ||||||
Spag6l (Chr16)    781   AAGGACAAAGATGAATATGTGAAGAAAAATGCTTGCACCTTAATCAGAGAGATTGCAAAA  840

Spag6 (Chr2)      841   CATACACCAGAGCTCTCGCAGCTGATTGTTAATGCCGGAGGTGTGGCTGCCGTGATCGAC  900
                        || || || |||||||||||||||||||||||||| ||||| |||||||||||||||||
Spag6l (Chr16)    841   CACACGCCTGAGCTCTCGCAGCTGATTGTTAATGCAGGAGGCGTGGCTGCCGTGATCGAT  900

Spag6 (Chr2)      901   TGCATCGGGTCCTGCAAAGGGAACATACGGCTGCCTGGCATCATGATGCTGGGTTACATG  960
                        ||||| ||||||||||||||||||||||||||||||||||||||||||||||||||| ||
Spag6l (Chr16)    901   TGCATTGGGTCCTGCAAAGGGAACATACGGCTGCCTGGCATCATGATGCTGGGTTACGTG  960

Spag6 (Chr2)      961   GCTGCTCATTCTGAGAACCTGGCCATGGCGGTGATCATCTCCAAGGGTGTACCCCAGTTG  1020
                        ||||||||||||||||||||||||||||||||||||||||||||||||||||||||||||
Spag6l (Chr16)    961   GCTGCTCATTCTGAGAACCTGGCCATGGCGGTGATCATCTCCAAGGGTGTACCCCAGTTG  1020

Spag6 (Chr2)      1021  TCAGACTGCCTGTCAGAAGAACCGGAAGATCATATTAAGGCTGCGGCTGCCTGGGCTCTA  1080
                        |||  |||||||||||||||||| |||||||||||||||||||| |||||||||||  |
Spag6l (Chr16)    1021  TCAATCTGCCTGTCAGAAGAACCAGAAGATCATATTAAGGCTGCTGCTGCCTGGGCCTTG  1080

Spag6 (Chr2)      1081  GGGCAGGTTGGGAGGCACACTCCAGAACATGCTCGGGCTGTCGCCATCACAAACACGTTG  1140
                        |||||| | || || ||||||||||| |||||  ||||||| ||  ||||||||||| ||
Spag6l (Chr16)    1081  GGGCAGCTGGGAAGACACACTCCAGAGCATGCCAGGGCTGTGGCTGTCACAAACACGCTG  1140

Spag6 (Chr2)      1141  CCCGTGCTGCTGGCTTTGTACATGTCCCCAGAGAGCTCTGAGGACCTGCAGGTGAAAAGT  1200
                        || || |||||| ||||||||||||| |||||||||||||||||||| ||| | ||||||
Spag6l (Chr16)    1141  CCAGTCCTGCTGTCTTTGTACATGTCACCAGAGAGCTCTGAGGACCTACAGCTCAAAAGT  1200

Spag6 (Chr2)      1201  AAAAAGGCCATAAAGAATATCATCCAAAAATGCACCTACCTCCCGGCGCTTGAGCCGTTT  1260
                        ||||| ||||||||||||||| | || ||||||||||||||||| || |||||||||||
Spag6l (Chr16)    1201  AAAAAAGCCATAAAGAATATCCTTCAGAAATGCACCTACCTCCCAGCCCTTGAGCCGTTC  1260

Spag6 (Chr2)      1261  CTCTATGATGCGCCTCCCAATATCCTGAAGTATGTCGCTGGACAGTTCAGTAAGGTGCTG  1320
                        || || || || |||||||||||||||||  |||| | ||| ||||||||||||||||||
Spag6l (Chr16)    1261  CTGTACGACGCACCTCCCAATATCCTGAAACATGTTGTTGGGCAGTTCAGTAAGGTGCTG  1320

Spag6 (Chr2)      1321  CCACATGACAGCAAAGCTCGTCGGCTTTTTGTAACAAGTGGTGGACTTAAAAAGATCCAA  1380
                        ||||||||||||||||| || |||||||| || || |||||||||||||||||| | ||
Spag6l (Chr16)    1321  CCACATGACAGCAAAGCCCGGCGGCTTTTCGTGACTAGTGGTGGACTTAAAAAGGTTCAG  1380

Spag6 (Chr2)      1381  GAGATCAAAGCAGAACCTGGTTCCATCCTCCAAGAGTATATCAACAACATCAACAGCTGC  1440
                        ||||| || |||||||| || ||  |||| || || || ||||||| ||||||||  ||
Spag6l (Chr16)    1381  GAGATAAAGGCAGAACCAGGCTCTCTCCTGCAGGAATACATCAACAGCATCAACAATTGT  1440

Spag6 (Chr2)      1441  TACCCAGAGGAAATAGTAAGGTACTATTCTCCTGGATACTCAGACATACTTCTGCAGAGG  1500
                        ||||||||||||||||||||||| ||||| ||||||||||| || | |||||||||||||
Spag6l (Chr16)    1441  TACCCAGAGGAAATAGTAAGGTATTATTCACCTGGATACTCCGATACACTTCTGCAGAGG  1500

Spag6 (Chr2)      1501  ATCGACAGCTATCAGCCTCTTATTAACTGAACGAAGTATA---TTATAATACTCAGATTC  1557
                         | |||||||| || || ||       ||||| ||| |||   ||||  | ||| | ||
Spag6l (Chr16)    1501  GTGGACAGCTACCAACCACT------GTGAACAAAGCATATTTTTATTCTCCTCGGTTTT  1554

Spag6 (Chr2)      1558  ACAGGAGAGTAAGGATAACAATTGTTAA  1585
                         |||||||||  | ||||  ||||||||
Spag6l (Chr16)    1555  CCAGGAGAGTGTGAATAATGATTGTTAA  1582
```

Supplemental Figure S1. The ancient *Spag6* gene shares high homology with the evolved gene, *Spag6l*.

Spag6 and *Spag6l* share high homology in nucleotide sequences. The coding sequences of the two genes were compared. Nucleotide sequence comparison using Basic Local Alignment Search Tool (BLAST) demonstrates high similarity between the two genes.

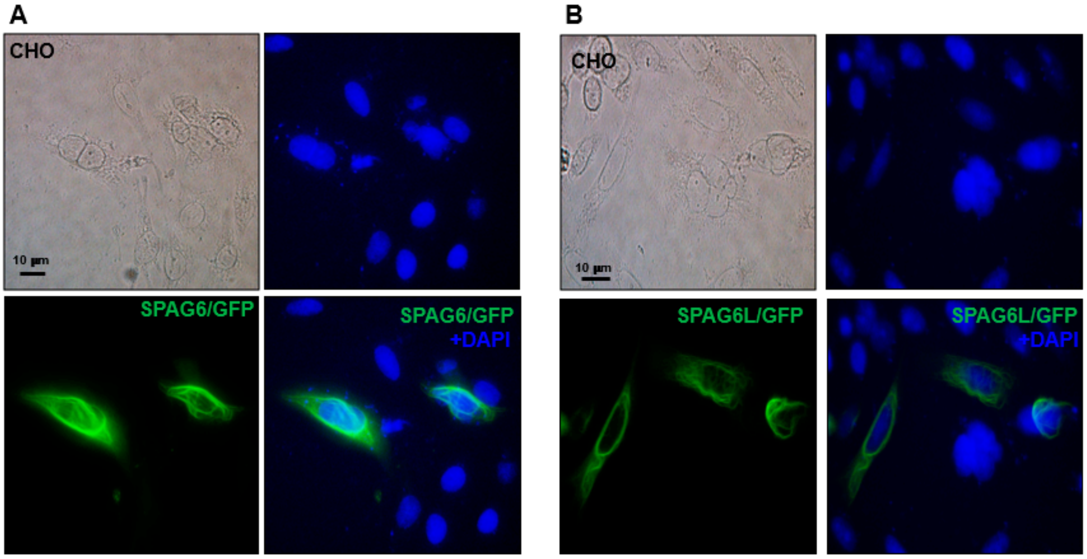

Supplemental Figure S2. Localization of SPAG6L/GFP in transfected CHO cells.

The SPAG6/pEGFP-N_2 (A) and SPAG6L/pEGFP-N_2 (B) plasmids were transfected into CHO cells. 48 hrs after transfection, the cells were fixed with 4% paraformaldehyde and mounted with a medium containing DAPI. Images were taken with a fluorescence microscope.

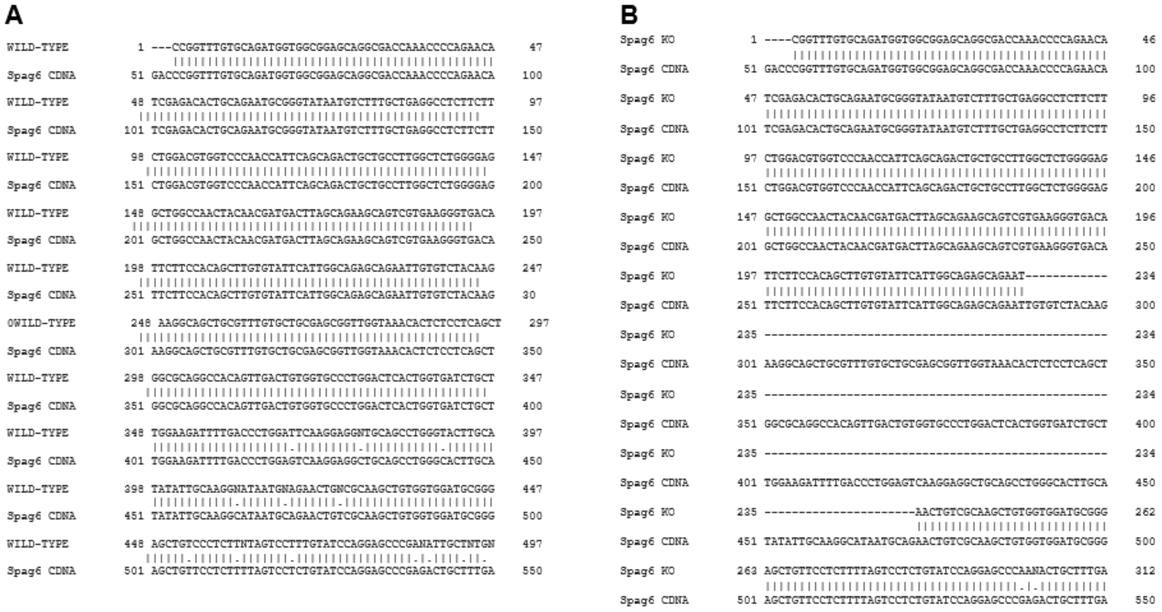

C

```
 R  F  V  Q  M  V  A  E  Q  A  T  K  P  Q  N  I  E  T  L  Q  N  A  G  I  M  S
CGGTTTGTGCAGATGGTGGCGGAGCAGGCGACCAAACCCCAGAACATCGAGACACTGCAGAATGCGGGTATAATGTCT
 L  L  R  P  L  L  L  D  V  V  P  T  I  Q  Q  T  A  A  L  A  L  G  R  L  A  N
TTGCTGAGGCCTCTTCTTCTGGACGTGGTCCCAACCATTCAGCAGACTGCTGCCTTGGCTCTGGGGAGGCTGGCCAAC
 Y  N  D  D  L  A  E  A  V  V  K  G  D  I  L  P  Q  L  V  Y  S  L  A  E  Q  N
TACAACGATGACTTAGCAGAAGCAGTCGTGAAGGGTGACATTCTTCCACAGCTTGTGTATTCATTGGCAGAGCAGAAT
 E  Q  N  N  C  R  K  L  W  W  M  R  E  L  F  L  F  -  S  S  V  S  R  S  P  X
AACTGTCGCAAGCTGTGGTGGATGCGGGAGCTGTTCCTCTTTTAGTCCTCTGTATCCAGGAGCCCAANACTGCTTTGA
```

Supplemental Figure S3. Examination of exon 4 deletion in *Spag6* KO mice by Sanger sequencing.

The full-length mouse *Spag6* coding regions were amplified by RT-PCR using testicular cDNA from a control mouse and a *Spag6* KO mouse. The two PCR products were sequenced using the forward primer, and the sequencing results were compared to the coding sequence of the mouse *Spag6* cDNA (GenBank: NM_001001334.2). A. BLAST results using the PCR product from the control mouse; B. BLAST results using the PCR product from the *Spag6* KO mouse. Notice that the control mouse contained an intact exon 4 sequence, but exon 4 was deleted in the KO mouse. C. Analysis of the amino acid sequence of the *Spag6* KO mice. The EMBOSS TranSeq program was used to analyze the amino acid sequence of the *Spag6* KO cDNA. Deletion of exon 4 of mouse *Spag6* resulted in a premature stop codon.

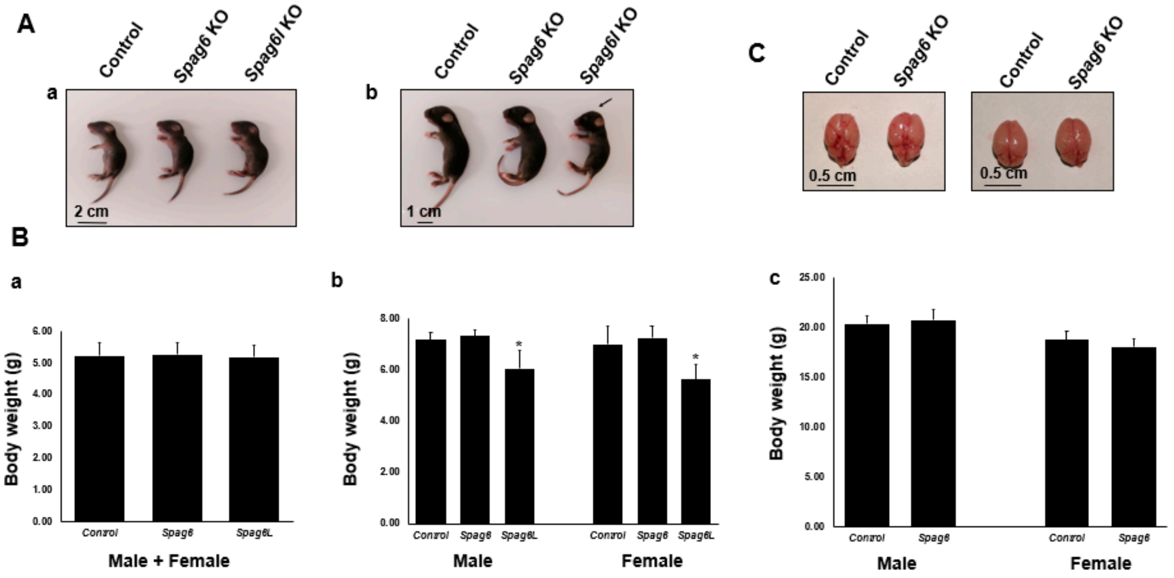

Supplemental Figure S4. Normal development of *Spag6* KO mice.

A. Representative images of the mice at the indicated ages. Notice that no difference was observed between the control and the *Spag6* KO mice at any of age analyzed. However, the *Spag6l* KO mice

were smaller at two weeks of age. a. One-week-old mice; b. Two-week-old mice. The arrow points to the head of a two-week-old *Spag6l* KO mouse with hydrocephalus.
B. Body weight of the control and *Spag6* KO mice at the indicated ages. Notice that there was no significant difference between the two groups. However, the body weight of the *Spag6l* KO mice was significantly lower at two weeks after birth. n=6. * P<0.05 compared to the control mice.
C. Representative images of brains of two-month-old control and *Spag6* KO mice. Hydrocephalus was not identified in any of the mice analyzed in this study.

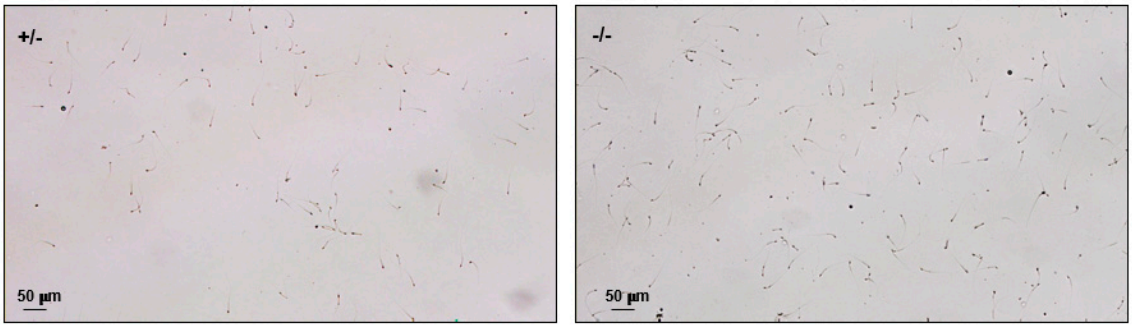

Supplemental Figure S5. Morphological examination of epididymal sperm of *Spag6* KO mice by light microscopy at low magnification.
Representative images of sperm at low magnification. Sperm density is similar between the control and the *Spag6* KO mice at the same dilutions.

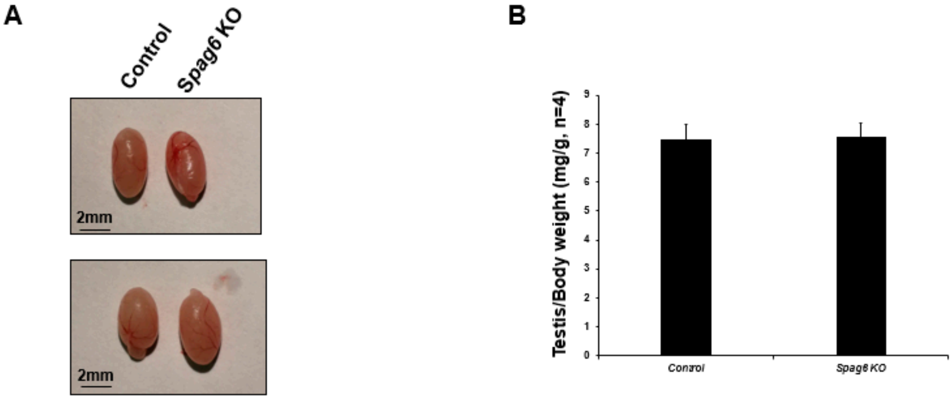

Supplemental Figure S6. *Spag6* KO mice have normal testis weight.
A. Representative image showing two pairs of testes from 4-month-old *Spag6* KO mice and their littermates;
B. A statistical analysis to compare the testis weight/body weight between the control and the *Spag6* KO mice. n=4. No significant difference was found (P>0.05).

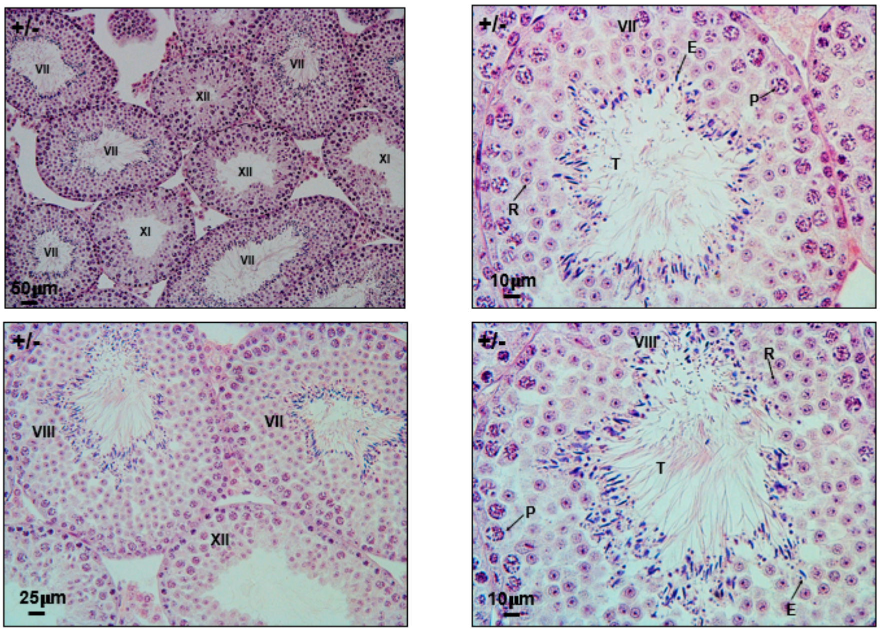

A

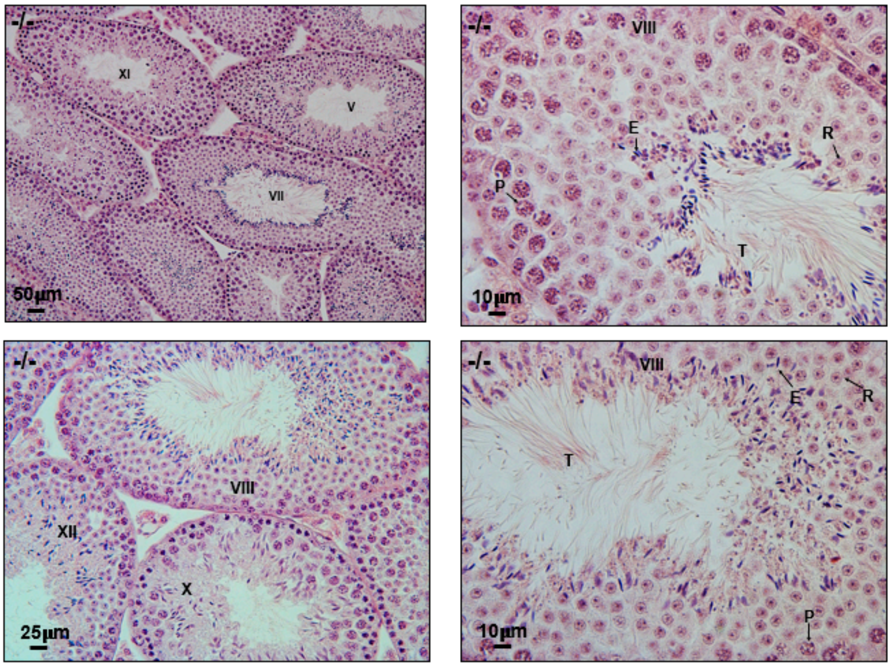

B

Supplemental Figure S7. Low and high magnification of testis histology.

Notice that both the control (A) and the *Spag6* KO (B) mice show normal seminiferous tubule structure at low (left) and high (right) magnification. P, pachytene spermatocytes, R, round spermatids, E, elongating spermatids, T, tail of sperm being released.

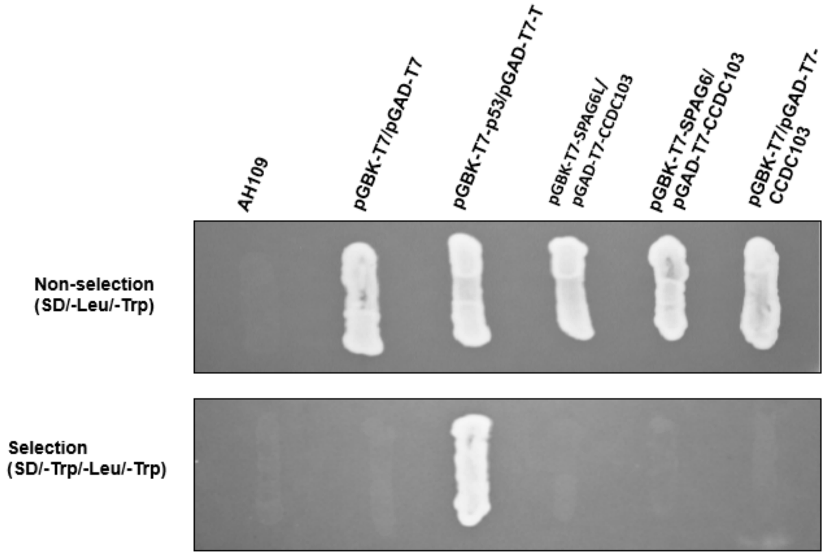

Supplemental Figure S8. The two mouse SPAG6 proteins do not bind to CCDC103.
Direct yeast two-hybrid assay to analyze interactions between the two mouse SPAG6 proteins and CCDC103. Neither of the two SPAG6 proteins bind to CCDC103.
